# Interactions between Fungal-Infected *Helicoverpa armigera* and the Predator *Chrysoperla externa*

**DOI:** 10.3390/insects10100309

**Published:** 2019-09-20

**Authors:** Pamella Mingotti Dias, Elisângela de Souza Loureiro, Luis Gustavo Amorim Pessoa, Francisco Mendes de Oliveira Neto, Ricardo Alexandre de Souza Tosta, Paulo Eduardo Teodoro

**Affiliations:** 1 Graduate Program in Entomology and Biodiversity Conservation (PPGECB), Universidade Federal da Grande Dourados, Dourados MS 79.804-970, Brazil; elisangela.loureiro@ufms.br; 2 Agronomy Universidade Federal of Mato Grosso do Sul (CPCS), Chapadão do Sul MS 79.560-000, Brazil; luis.pessoa@ufms.br (L.G.A.P.); mendesfrancisco858@gmail.com (F.M.d.O.N.); ricardoagronomia2014@gmail.com (R.A.d.S.T.); paulo.teodoro@ufms.br (P.E.T.)

**Keywords:** lepidoptera, Hypocreales, biological control, green lacewing, integrated pest management, compatibility

## Abstract

The aim of the present study was to evaluate the interactions between *Chrysoperla externa* (Hagen, 1861) and the eggs and first-instar larvae of *Helicoverpa armigera* (Hübner 1805) infected by entomopathogenic fungi. The *H. armigera* eggs and larvae were treated with sterile distilled water + 0.01% Tween 80 (T1, control), *Beauveria bassiana* (Bals.) Vuill (T2), *Metarhizium anisopliae* (Metsch.) Sorok (T3), or *Metarhizium rileyi* (Farlow) Samson. (T4) at different concentrations (1 × 10^7^, 1 × 10^8^, and 1 × 10^9^ con. mL^−1^). For each treatment, a single third-instar *C. externa* was offered prey (a combination of 80 eggs and 50 first-instar *H. armigera* larvae) at 0, 24, and 48 h after inoculation. Ten trials were completed for each treatment, and the entire experiment was repeated three times. Neither the concentrations of fungi nor the application method affected consumption by *C. externa*. Because all the predator larvae reached the pupal phase, with 100% viability in adults, these results suggest that entomopathogenic fungi and *C. externa* are compatible and that the simultaneous use of these biological control agents is possible for managing *H. armigera*.

## 1. Introduction

The occurrence of lepidopteran pests at high population densities has led to major economic losses in the agricultural and forestry sectors of Brazil [1]. The cotton caterpillar *Helicoverpa armigera* (Hübner 1805) (Lepidoptera: Noctuidae) was considered in Brazil, a quarantine pest, with reports during occurrence and major damage of the 2013/2014 agricultural year [2]. *H. armigera* has a high potential to become a pest in countries into which it enters because the adults are capable of migration and possess high reproductive potential [3,4], whereas the larvae exhibit aggressive, polyphagous, and destructive eating habits, indiscriminately consuming both the vegetative and reproductive structures (e.g., flowers, pods, and fruits) of a variety of agricultural crops [5,6,7]. Furthermore, the succession and crop rotation systems used in Brazil promote damage by *H. armigera* by providing abundant food resources [8].

The interactions between populations from different regions [9], poorly structured pest management programs, and indiscriminate insecticide use have contributed to the selection pressure that has led *H. armigera* populations to develop resistance to approximately 49 active ingredients of commercially available insecticides [10,11]. Effective integrated pest management (IPM) must involve a well-architectured structure that includes a variety of tools, including biological control agents, entomopathogenic fungi, and insect predators [12,13,14,15,16]. Confirmatory studies of pathogenicity and virulence of entomopathogenic fungi on *H. armigera* have been presented [17] using *Beauveria bassiana* (Bals.) Vuill [18], *Metarhizium anisopliae* (Metsch.) Sorok [19,20] and *Metarhizium rileyi* (Farlow) Samson [21].

Environmental factors such as temperature and precipitation can negatively affect the pathogenicity, virulence, and survival of entomopathogenic fungi. The combination of entomopathogenic fungi and other control agents such as predators may be a viable alternative in pest management [22]. Among entomophagous insects, the green lacewing, *Chrysoperla externa* (Hagen, 1861) (Neuroptera: Chrysopidae), a veracious predator that is highly abundant and easy to culture in the laboratory, has gained attention in research into biological control [23,24].

The efficacy of entomopathogenic fungi depends on their high virulence, in regard to arthropod pests, as well as on their selectivity and low virulence toward non-target insects [25]. Studies in Brazil have demonstrated the compatibility of fungal and lacewing species [26,27,28,29], and Chrysopidae have also been demonstrated as effective consumers of lepidopteran eggs [30,31,32,33], including those of *H. armigera* [34]. However, there is still much to learn about the effect of entomopathogenic fungi on the green lacewing, especially regarding its consumption of infected prey.

Studies that have investigated the direct and indirect effects of entomopathogens on predators and parasitoids have mostly focused on the direct application of fungal suspensions and have generally reported that higher doses are correlated with greater negative effects [35].

The genera *Beauveria* and *Metarhizium* may present endophytic action on plants providing better physiological performance and reducing the population arthropod-pests through synergism between predators and parasitoids [35,36]. It has been reported by [37,38] that the interaction between entomopathogenic fungi and the predator of *Chrysoperla carnea* (Neuroptera: Chrysopidae) is beneficial, thus enabling the joint application of these biocontrol organisms in aphid management.

The objective of the present study was to evaluate the interaction between *C. externa* and the eggs and first-instar larvae of *H. armigera* treated with different concentrations of *B. bassiana* (isolate ESALQ PL63), *M. anisopliae* (isolate ESALQ E9), or *M. rileyi* (isolate UFMS 03).

## 2. Materials and Methods

### 2.1. Collection and Rearing of H. armigera

During the 2018–2019 growing season, *H. armigera* adults and larvae of different ages were collected from soybean fields at the Universidade Federal de Mato Grosso do Sul (Campus of Chapadão do Sul, MS, Brazil) that had not been sprayed with chemicals. In the laboratory, the *H. armigera* larvae were transferred to plastic containers (16 insects per container; 25 ± 1 °C, 70 ± 10% RH (relative humidity), and photoperiod 12 h day and 12 h night and daily fed an artificial diet mainly composed of white beans adapted from [39]. The *H. armigera* pupae were separated by sex and, were assembled into male–female pairs, totaling sixteen adults per cage, using methods [40,41].

### 2.2. Collection and Rearing of C. externa

Adults of *C. externa* were identified from collections made in the same area mentioned above in and using the method [42], and were later transferred to an acclimatized room (25 ± 1 °C, 70 ± 10% RH, and photoperiod of 12 h day and 12 h night). Within the room, the collected adults were kept in PVC (Polyvinyl chloride) cages (height 23 cm, diameter 10 cm) that were lined with white bond paper (for oviposition), with the lower end supported by styrofoam lined with a paper towel and the upper end sealed with “VOILE fabric”. An artificial diet of beer yeast and honey [43] was provided using an adapted feeder, which was composed of a soft sponge containing paste-shaped diet over the top base and the bottom part inserted into a 10-mL cylindrical tube with distilled water. This adaptation allowed the sponge to be constantly humidified by constantly providing water and food. At 24 h after oviposition, eggs were removed from the oviposition substrate using either scissors or a fine plastic comb. The eggs (10 each) were then transferred to 500-mL plastic containers that had been properly sanitized using 70% alcohol and 12 h of UV irradiation. The containers (i.e., cages) contained strips of paper to create a refuge for the larvae against cannibalism. Larvae were fed with eggs of *Anagasta kuehniella* (Zeller) (Lepidoptera: Pyralidae) daily until sufficient numbers of larvae were available for bioassays [34].

### 2.3. Fungal Isolates

*Beauveria bassiana* (isolate ESALQ PL63) and *M. anisopliae* (isolate ESALQ E9) were extracted from the commercial mycoinsecticides BOVERIL^®^ and METARRIL^®^, respectively (Koppert Biological Systems, Piracicaba, SP, Brazil), whereas *Metarhizium rileyi* (UFMS 03) was obtained from the entomopathogen bank of the Entomology Laboratory of the Universidade Federal de Mato Grosso do Sul (Campus of Chapadão do Sul, MS, Brazil). However, because no commercial formulas contain *M. rileyi* (UFMS 03), this isolate was produced using Sabouraud media and previously reported methods (25 ± 1 °C, 70 ± 10% RH, and photoperiod of 12 h day and 12 h night) [44]. To prepare fungal suspensions, the isolates were diluted using sterile distilled water that contained 0.01% (*v*/*v*) Tween80^®^, and the conidia were counted using a Neubauer chamber to standardize the concentrations [45].

### 2.4. Interaction between C. externa and Fungi-Treated H. armigera

The bioassay was performed in the laboratory under the following conditions: (25 ± 1 °C, 70 ± 10% RH, and photoperiod of 12 h day and 12 h night). Two prey stages, (eggs and caterpillars) were used in separate experiments. 

For the egg bioassay, each *C. externa* larvae received 80 *H. armigera* eggs (>12 h). In the caterpillar bioassay, each *C. externa* larvae received 50 first-instar *H. armigera* larvae (1 to 3 mm long). These preys were made available to the predator in 250 mL plastic containers that were properly sanitized with 70% alcohol and 12 h of UV irradiation.

The *H. armigera* eggs and larvae were treated with sterile distilled water + 0.01% Tween 80 (T1, control), *B. bassiana* (T2), *M. anisopliae* (T3), or *M. rileyi* (T4) at different concentrations (1 × 10^7^, 1 × 10^8^, and 1 × 10^9^ con. mL^−1^), and a control treatment was included for each exposure time (0, 24 and 48 h) and using either direct application (D.A.) or dry film (D.F.) application methods. The treatments were applied using an adapted Potter Tower, with a pressure of 1.5 MPa. For the direct application, fungal suspensions and control treatment were applied directly to eggs, larvae and plastic containers [46] after the dry surface (time zero), with release of *C. externa*, whereas the dry film method was applied only in the containers, with transfer of eggs and larvae of *H. armigera* to the plastic container containing the larvae of *C. externa* after drying the excess moisture [47]. The protocol proposed by the International Organization for Integrated Biological and Control of Harmful Animals and Plants, Western Palaearctic Regional Section was used to standardize selectivity tests for entomopathogens [48].

For each treatment, a single third-instar larvae of *C. externa*, after 24 h of fasting, was offered prey (80 eggs and 50 first-instar *H. armigera* larvae) at 0, 24, and 48 h after inoculation. The choice of this predator instar refers to the most voracious larval age. In the third instar, green lacewing can reach 72–85% prey consumption equivalent to the predatory capacity of the entire larval phase, depending on prey type [49].

After the start of each test, the containers were sealed with plastic film and plastic caps with small ventilation holes and stored in a biological oxygen demand (B.O.D) chamber under the following conditions: 25 ± 1 ° C, 70 ± 10% RH, and photoperiod of 12 h day and 12 h night. The performance of *C. externa* was evaluated over a 24-h period, and performance was assessed on the basis of mean number of eggs and larvae consumed, ability to detect infected prey, and the interactive behaviors of both the predators and prey.

In order to evaluate the conidia recovery, three inoculated insects from each replicate were washed using sterile distilled water with 0.01% Tween80^®^, and then the number of conidia was estimated using a Neubauer counting chamber, (Boeco^®^), Lab-Líder, Ribeirão Preto, SP, Brazil.

We performed three replicates of the bioassay of the interaction between *C. externa* and inoculated *H. armigera* eggs and larvae.

### 2.5. Statistical Analysis

The present study involved a completely randomized experimental design (DIC) that included factorial arrangement (4 × 3 × 2), with four treatments (P), three suspension concentrations (C), and two application methods (A). Each trial included a third-instar *C. externa* larva, 80 *H. armigera* eggs, and 50 first-instar *H. armigera* larvae, and 10 replicates were performed for each combination of treatment, suspension concentration, and application method.

The consumption of inoculated *H. armigera* eggs and larvae after 0, 24, 48, and 72 h was normally distributed. Therefore, the data were subjected to analysis of variance (ANOVA), and means were compared using the Tukey test, at 5% probability, using Rbio [50] and Genes [51].

## 3. Results

### 3.1. Interaction between C. externa and Fungi-Treated H. armigera Eggs

Neither product (P), application method (A), suspension concentration (C), or interactions between these factors (A × P, A × C, P × C, or A × P × C) significantly affected the consumption of *H. armigera* eggs by *C. externa,* regardless of exposure time (*p* > 0.05; Table 1), and the *C. externa* larvae consumed statistically equivalent numbers of *H. armigera* eggs, regardless of treatment (vs. control), product, application method, suspension concentration, or exposure time (Table 2).

### 3.2. Interaction between C. externa and Fungi-Treated H. armigera Larvae

Neither product (P), application method (A), suspension concentration (C), or interactions between these factors (A × P, A × C, P × C, or A × P × C) significantly affected the consumption of *H. armigera* larvae by *C. externa*, regardless of exposure time (*p* > 0.05; Table 3), and the *C. externa* larvae consumed statistically equivalent numbers of *H. armigera* eggs, regardless of treatment (vs. control), product, application method, or suspension concentration (Table 4).

### 3.3. Effect of Entomopathogenic Fungi on Predator Behavior

It was clear that the *C. externa* larvae were not killed by consuming entomopathogen-infected *H. armigera* eggs or larvae, at least not within the first several days after consumption (Figure S1A). However, the *H. armigera* first instar caterpillars exhibited defense mechanisms that prevented the larvae of *C. externa* from eating them for a period. The larvae were observed to cluster in the area that was opposite the predator and to produce webs, which, in many cases, allowed them to defend themselves against predation by *C. externa*. The predator, in turn, remained on the opposite side of the arena when a caterpillar defense was mounted.

The *C. externa* larvae returned to searching for food and capturing prey, preferring to attack the posterior part of caterpillars and to grab them with their mouthparts (Figure S1B). Some caterpillars managed to escape, but many of those were still debilitated by a loss of fluids, owing to injuries sustained from the attack. The caterpillars remained defensive and mobile in the early hours of evaluation but later exhibited reduced mobility, which allowed them to be captured (Figure S1C).

All the *C. externa* larvae reached the pupal stage, with 100% viability in adults.

## 4. Discussion

The present study makes several useful observations, especially considering the scarcity of information on the potential of *C. externa* as an insect predator of *H. armigera* and on the interactions between *C. externa* and entomopathogenic fungi. The findings of the present study indicate that the interactions between *C. externa* and entomopathogenic fungi are positive because the fungi (*B. bassiana*, *M. anisopliae*, and *M. rileyi*) do not interfere with the ability of *C. externa* to consume *H. armigera* larvae, assuming that the entomopathogens do not influence the nutritional quality of the prey.

Entomopathogenic fungi are a primary source of metabolites and exhibit a variety of antimicrobial, insecticidal and cytotoxic activities [52]. Through chemical communication, these metabolites can be recognized by insects as predators that can reduce feeding by identifying prey infected with hyphae, blastospores after conidiogenesis due to the toxic metabolic action of entomopathogenic fungi [53].

This research assumed that *C. externa* consumption was not disrupted when prey were infected by entomopathogenic fungi. However, studies with the predator *Orius insidiosus* (SAY, 1832) (Hemiptera, Anthocoridae) shown that this predator can identify infected aphids and starve to death before consuming the infected prey [54].

Even though there was no significant difference between the mean numbers of eggs consumed by third-instar green lacewing, small decreases were noted in the consumption of eggs by *C. externa* at 48–72 h after treatment. This suggests that: (1) the prolonged period of fungal contact with the eggs enabled the germination of the fungus, thereby making the prey unappetizing to the predator, (2) the eggs were more nutritious at this time, owing to the stage of embryo formation, and satiated the predator for a longer period, or (3) the eggs that were exposed to the predator for less time (0–24 and 24–48 h) released chemical propionic, benefit during the capture by the *C. externa* larvae. Previous studies have reported that *C. carnea* larvae benefit from the capture of *Helicoverpa zea* (Boddie) (Lepidoptera: Noctuidae) by the release of kairomones from eggs and adult scales [55,56,57].

There is a possibility of simultaneous use of entomopathogenic fungi and *C. externa* in the integrated management of lepidopteran pests, since the consumption rates of *C. externa* prey were not reduced by increasing concentrations of entomopathogen suspensions. It has been reported that high concentrations of entomopathogens when applied directly to entomophagous organisms may have a greater negative effect than lower concentrations [25].

Fungi act through direct contact and infect a broad spectrum of insect hosts [58]. Both the (D.A.) and (D.F.) methods increase the possibility of contact between insects and fungi in the field. In green lacewings, naked-type larvae have a habit of actively searching for food, moving with the mouthpieces partially open and parallel to the substrate to initiate predation more quickly upon direct contact with prey [59].

In the present study, *C. externa* larvae used all of the available space in the arenas, and, consequently, all the larvae came in contact with conidia, regardless of the application method. The dry film method is important because it has the potential to disperse conidia using other entomophagous organisms, as indicated by the recovery of conidia from the insects.

Ladybugs (Coccinellidae) and lacewings can naturally spread conidia, with reports that *Harmonia axyridis* (Pallas, l773) (Coleoptera: Coccinellidae) and *C. carnea* disseminated 93 and 89% of *B. bassiana* conidia, respectively, up to 2.4 m away from the site of release, with a positive interaction between infected larvae and aphid consumption.

In the present study, the consumption of infected prey and, thus, exposure to entomopathogenic fungi did not affect *C. externa* larval development, pupal development, or adult viability. The quality and quantity of food consumed by the predator during the first larval instars directly influence adult viability, where the highest food intake of lacewings is during the third instar consisting of approximately 72–85% of consumption during the juvenile stages, depending on prey species [49].

When larvae *H. armigera* feel threatened by the presence of other insects, they bend with the cephalic capsule towards the ventral region of the first pair of false legs, probably as a defense mechanism. This behavior was noticeable during the approach of the larvae *C. externa*; the caterpillars exhibited cephalic capsule curvature, cobweb production and crowding, with collective defense.

These behavioral characteristics may be the result of evolutionary adaptations and population mixing, which makes them more aggressive [2,5,9,60]. The insects can develop defense mechanisms against entomopathogens, predators, and parasitoids, and such mechanisms can include recognition, agglutination, and the activation of proteolytic enzymes, which cause hemolymph coagulation, melanin production, cellular reactions, and the synthesis of antimicrobial peptides and protease inhibitors [61,62].

Studies of the interactions between microbial agents and predators are valid because entomopathogenic fungi produce a wide range of pathogenic metabolites and are capable of infecting several orders of insects [63]. According to [64], entomopathogenic fungi may also affect host development, fitness, physiology, and behavior.

The compatibility observed in the present study suggests the joint action of *B. bassiana*, *M. anisopliae*, *M. rileyi* and *C. externa* in the integrated management of *H. armigera*. It is important to validate the action of natural enemies on lepidopteran-pest eggs, the reduction in the number of pest generations. This study corroborates the results of previous studies in Brazil [30,60] that demonstrated the effectiveness of *C. externa* predation on *H. armigera* eggs and first-instar larvae.

Investigated the interactions of entomopathogenic fungi with *C. externa* and consumption capacity of this predator in relation to *H. armigera*, verifying the general predation and compatibility between the tested fungi. We suggest that future studies evaluate the interactions between *C. externa* and other microbial agents and investigate the applicability of such microorganisms and entomophages used in the integrated management of lepidopteran pests in important crops.

## 5. Conclusions

The consumption of *H. armigera* eggs and first-instar larvae by *C. externa* was not reduced by the application of entomopathogenic fungi (*B. bassiana*, *M. anisopliae*, or *M. rileyi*), regardless of concentration (1 × 10^7^, 1 × 10^8^, or 1 × 10^9^ con. mL^−1^) or application method (DA or DF). *Chrysoperla externa* also failed to exhibit discriminatory potential in relation to the contaminated prey at 0, 24, or 48 h after inoculation. These findings indicate that there was a positive interaction between the entomopathogenic fungi and the third-instar larvae of *C. externa* and that their simultaneous use may be helpful for controlling *H. armigera* populations. All the *C. externa* larvae reached the pupal stage, with 100% adult viability.

## Figures and Tables

**Table 1:** ANOVA table for the interactions between the factors studied and the consumption of eggs of *H. armigera* in different times (0, 24, 48, and 72 h) of exposure to *C. externa* (T: 25 ± 1 °C, RH of 70 ± 10% and photoperiod of 12 h day and 12 h night).

SV	DF	0–24 h	24–48 h	48–72 h
Application (A)	1	1.62 ^ns^	3.12 ^ns^	231.12 ^ns^
Product (P)	3	1.84 ^ns^	14.54 ^ns^	68.00 ^ns^
Concentration (C)	3	2.27 ^ns^	1.41 ^ns^	7.49 ^ns^
A × P	3	10.86 ^ns^	1.51 ^ns^	31.55 ^ns^
A × C	3	4.67 ^ns^	0.34 ^ns^	6.78 ^ns^
P × C	9	7.65 ^ns^	1.18 ^ns^	7.59 ^ns^
A × P × C	9	0.69 ^ns^	1.60 ^ns^	6.85 ^ns^
Residue	180	62.36 ^ns^	78.11 ^ns^	78.29 ^ns^

ns: not-significance at 5% probability by F-test. SV: source of variation; DF: degrees of freedom; Numbers are F-values.

**Table 2:** Mean consumption (mean ± standard error) of eggs of *H. armigera* infected with *B. bassiana and M. anisopliae* and *M. rileyi* in times (0, 24, 48 and 72 h) of exposure to *C. externa* (T: 25 ± 1 °C, RH of 70 ± 10%, and photoperiod of 12 h day and 12 h night).

Application (A)	0–24 h	24–48 h	48–72 h
Direct (D.A.)	72.28 ± 0.86 a	71.01 ± 0.82 a	68.51 ± 0.84 a
Dry film (D.F.)	72.10 ± 0.63 a	70.76 ± 0.85 a	66.36 ± 0.88 a
**Product (P)**			
Control	73.50 ± 1.19 a	72.25 ± 1.69 a	69.30 ± 3.16 a
*B. bassiana*	72.20 ± 3.19 a	70.61 ± 4.00 a	66.41 ± 4.04 a
*M. anisopliae*	71.56 ± 3.71 a	70.88 ± 2.54 a	68.38 ± 2.87 a
*M. rileyi*	72.36 ± 2.55 a	70.70 ± 3.91 a	66.88 ± 3.35 a
**Concentration (C)**			
10^7^ con. mL^−1^	72.10 ± 1.17 a	70.95 ± 1.14 a	67.13 ± 1.15 a
10^8^ con. mL^−1^	71.78 ± 0.90 a	70.61 ± 0.95 a	66.85 ± 1.06 a
10^9^ con. mL^−1^	72.25 ± 0.93 a	70.63 ± 1.25 a	67.70 ± 1.07 a

For each factor (application, product or concentration), means followed by letters equal in the same column do not differ statistically by Tukey test at 5% probability.

**Table 3:** ANOVA table for the interactions between the factors studied and the consumption of larvae of *H. armigera* in different times (0, 24, 48 and 72 h) of exposure a *C. externa* (T: 25 ± 1 °C, RH of 70 ± 10% and photoperiod of 12 h day and 12 h night).

SV	GL	0–24 h	24–48 h	48–72 h
Application (A)	1	32.00 ^ns^	3.12 ^ns^	0.04 ^ns^
Product (P)	3	34.30 ^ns^	8.80 ^ns^	2.34 ^ns^
Concentration (C)	3	20.27 ^ns^	0.77 ^ns^	0.41 ^ns^
A × P	3	1.46 ^ns^	0.45 ^ns^	0.30 ^ns^
A × C	3	20.07 ^ns^	1.34 ^ns^	2.23 ^ns^
P × C	9	3.02 ^ns^	4.34 ^ns^	0.69 ^ns^
A × P × C	9	10.62 ^ns^	0.56 ^ns^	0.85 ^ns^
Residue	180	26.41 ^ns^	0.41 ^ns^	18.56 ^ns^

ns: not-significance at 5% probability by F-test. SV: source of variation; DF: degrees of freedom; Numbers are F-values.

**Table 4:** Consumption (mean ± standard error) of larvae of *H. armigera* infected with *B. bassiana*, *M. anisopliae* and *M. rileyi* in times (0, 24, 48 and 72 h) of exposure a *C. externa* (T: 25 ± 1 °C, RH of 70 ± 10% and photoperiod of 12 h day and 12 h night).

Application (A)	0–24 h	24–48 h	48–72 h
Direct (D.A.)	45.54 ± 0.68 a	46.54 ± 0.33 a	45.38 ± 0.40 a
Dry film (D.O.)	45.58 ± 0.52 a	46.74 ± 0.31 a	45.29 ± 0.44 a
**Product (P)**			
Control	47.75 ± 0.40 a	47.70 ± 0.60 a	45.95 ± 1.15 a
*B. bassiana*	45.53 ± 2.78 a	46.63 ± 1.32 a	45.13 ± 1.69 a
*M. anisopliae*	44.58 ± 3.25 a	46.26 ± 1.52 a	45.35 ± 1.71 a
*M. rileyi*	45.83 ± 1.39 a	46.66 ± 1.30 a	45.31 ± 1.90 a
**(C) concentration**			
10^7^ con. mL^−1^	45.88 ± 0.74 a	46.55 ± 0.44 a	45.25 ± 0.50 a
10^8^ con. mL^−1^	45.11 ± 0.91 a	46.48 ± 0.43 a	45.28 ± 0.59 a
10^9^ con. mL^−1^	44.95 ± 0.81 a	46.53 ± 0.44 a	45.26 ± 0.57 a

For each factor (application, product or concentration), means followed by letters equal in the same column do not differ statistically by Tukey test at 5% probability.

## Supplementary Materials

The following are available online at https://www.mdpi.com/2075-4450/10/10/309/s1, Figure S1: Capture and consumption of first-instar *Helicoverpa armigera* larvae by third-instar *Chrysoperla externa* larvae (time zero). (**A**) catch and suction of cellular fluid of caterpillars with 2 hours of evaluation; (**B**) Capture of larvae by the final portion of the abdomen with a period of 6 hours of evaluation; (**C**) cephalic capsules of caterpillars ingested by the green lacewing during 24 hours of evaluation. (Photos registered with 13 Mpx camera and aid of stereoscopic microscope with an increase of 20 ×).
