# Supplementary material for: Interactions between Fungal-Infected Helicoverpa armigera and the Predator Chrysoperla externa
Source: Insects. 2019 Sep 20;10(10):309. doi: 10.3390/insects10100309 (PMC6835894; doi:10.3390/insects10100309)
Supplement: Supplementary file 1 [file insects-10-00309-s001.pdf]

insects

Supplementary Materials

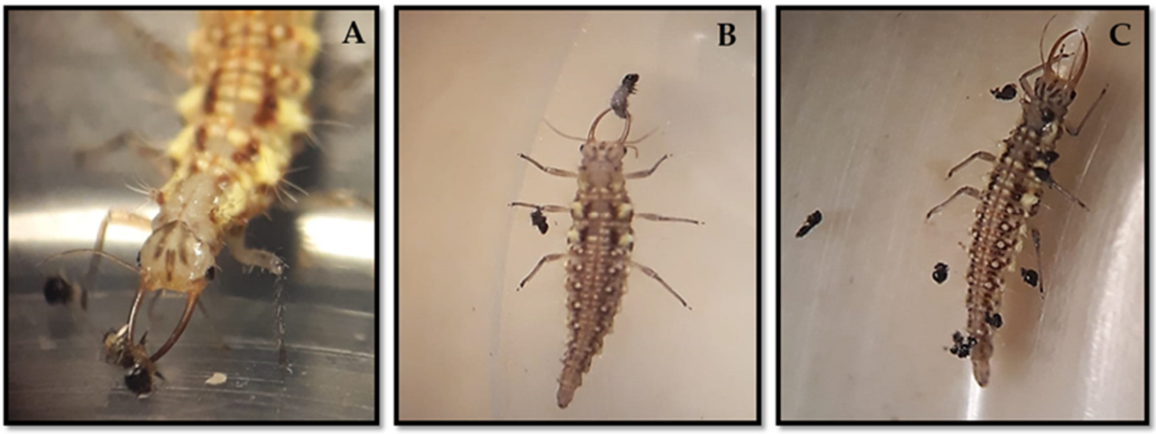

Figure S1. Capture and consumption of first-instar *Helicoverpa armigera* larvae by third-instar *Chrysoperla externa* larvae (time zero). (**A**) catch and suction of cellular fluid of caterpillars with 2 hours of evaluation; (**B**) Capture of larvae by the final portion of the abdomen with a period of 6 hours of evaluation; (**C**) cephalic capsules of caterpillars ingested by the green lacewing during 24 hours of evaluation. (Photos registered with 13 Mpx camera and aid of stereoscopic microscope with an increase of 20 ×).
